# Implementation of the patient safety incident guideline in district health services, Western Cape

**DOI:** 10.4102/safp.v67i1.6108

**Published:** 2025-04-30

**Authors:** Robert J. Mash, Kaashiefah Adamson, Abdul Isaacs, Gavin Hendricks, Jani Fouche, Jennie Morgan, Klaus von Pressentin, Lawson Eksteen, Leigh Wagner, Liezel Rossouw, Luke Profitt, Marshall Lockett, Milton Groenewald, Mumtaz Abbas, Paddy Gloster, Paul Kapp, Stefanie Perold, Tracey-Leigh Abrahams, Werner Viljoen

**Affiliations:** 1 Division of Family Medicine and Primary Care, Faculty of Medicine and Health Sciences, Stellenbosch University, Cape Town, South Africa; 2 Division of Family Medicine, Department of Family, Community and Emergency Care, Faculty of Health Sciences, University of Cape Town, Cape Town, South Africa; 3 Metro Health Services, Western Cape Department of Health and Wellness, Cape Town, South Africa; 4 Rural Health Services, Western Cape Department of Health and Wellness, Cape Town, South Africa

**Keywords:** patient safety, primary care, district hospitals, risk management, patient safety incidents

## Abstract

**Background:**

South Africa has implemented a patient safety incident reporting and learning system (PSIRLS) in 2022. The aim of this study was to evaluate the implementation of this PSIRLS in the district health services of the Western Cape.

**Methods:**

A convergent parallel mixed methods study was conducted within a practice-based research network. Qualitative data were collected through 15 semi-structured interviews with purposefully selected respondents from 10 district hospitals and 5 primary care facilities, and the data were thematically analysed. Quantitative data for 2023 were collected from the PSIRLS at 16 facilities and analysed descriptively.

**Results:**

The PSIRLS was adopted by all facilities. Overall, 577 patient safety incidents (PSI) were reported (range 0–148 per facility) with 91% from district hospitals, 18% severity assessment code 1 (SAC1), 33% caused harm and 72% in hospital wards. Staff were prompted to follow the steps by structured forms and the digital system. Patient safety incidents were reported by health professionals, although clinicians were concerned about blame and damaging teamwork. Severity assessment code 1 were reported on time (median < 24 h) and investigated promptly (median closure 4 days). Opportunity costs could be significant. While the system improved patient safety, it primarily focussed on behavioural interventions. Austerity measures and the reduction of quality assurance managers posed a threat to the system.

**Conclusion:**

Strengthening training for operational managers and clinical staff, enhancing infrastructure and addressing mental health-related incidents are crucial for long-term success. Future research should explore sustainable strategies to overcome financial and organisational barriers.

**Contribution:**

The need for continuous training, awareness and systemic improvements to enhance the effectiveness of PSIRLS in South African district health services.

## Introduction

The goal of universal health coverage includes a commitment to safe healthcare and the World Health Organization (WHO) acknowledged this in the resolution for ‘global action on patient safety’ at the World Health Assembly in 2019.^1^ Patient harm because of adverse events is one of the top 10 causes of death and disability in the world.^2^ At least five people die every minute because of unsafe care. In high-income countries, one in 10 patients is harmed while receiving hospital care.^3^ Approximately two-thirds of all patient harm occurs in low- and middle-income countries.^4^ Up to 15% of hospital expenditure may be a direct result of patient safety incidents (PSIs). It is estimated that, in primary and outpatient care, four out of every 10 patients may be harmed.^4^

A PSI is defined as ‘an unplanned or unintended event or circumstance that could have resulted or did result in harm to a patient while in the care of a health facility. This event is thus not because of the underlying health condition or the natural progression of the disease. An incident can be a near miss, no harm incident or harmful incident (adverse event)’.^5^ Such patient safety incident reporting and learning systems (PSIRLS) have been introduced in 70% of countries, although only 32% actively report PSIs in the majority of facilities.^6^ The African continent scores lowest of all regions of the world in the use of PSIRLS and digital technology, although South Africa has implemented such a system.^6^

Clinical governance ‘is a framework that helps managers and clinicians to improve the quality of their services and safeguard standards of care’.^7^ It has four key components: clinical effectiveness, patient safety, patient focus and continuing professional development. Patient safety includes risk management to identify risks and avoid patient harm, and the investigation of adverse events or actual harm to learn lessons and prevent them from occurring again.^8^ Therefore, there needs to be a system for identifying and reporting risks or adverse events that involve all facility members. The goal is to prevent injury, ensure patient safety and improve the quality of care. This can also be a cost-saving measure as it reduces the cost of litigation or injuries to staff.

The National Guideline for Patient Safety Incident Reporting and Learning in the Health Sector of South Africa was first published in 2018 and revised in 2022 and was developed in collaboration with the WHO.^5^ The purpose of the guideline is to ‘provide direction to the health sector of South Africa regarding the management of PSI reporting, including the provision of appropriate feedback to patients, families/support persons and clinicians, as well as the sharing of lessons learned to prevent patient harm’.^5^ The guideline categorises PSIs into four severity assessment codes (SAC):^5^

- SAC 1: Serious harm or death that is/could be specifically caused by healthcare rather than the patient’s underlying condition or illness.
- SAC 2: Moderate harm that is/could be specifically caused by healthcare rather than the patient’s underlying condition or illness.
- SAC 3: Minor harm that is/could be specifically caused by healthcare rather than the patient’s underlying condition or illness.
- SAC 4: No harm.

The guideline outlines the steps that should be taken to handle a PSI and the formation of a patient safety committee at the facility. Forms are also provided to report on the type of PSI, contributing factors and outcomes. A web-based PSIRLS was developed to support the guideline.

Although the number of reported PSI cases increased from 17 341 in 2018/2019 to 21 726 in 2020/2021, there is still only 51% compliance with the guidelines and variability between provinces.^5^ The case closure rate within 60 days is high (99%), although only 73% of SAC1 PSIs are reported within 24 h. The distribution of PSIs is SAC1 (23%), SAC2 (27%) and SAC3 (49%). In 2020/2021, the top three specific types of PSI were related to behaviour (27%) (e.g. sexual or physical assault by staff, patient or visitor), clinical processes and procedures (27%) and patient accidents (12%).

There are few studies on the implementation of PSI reporting and learning in the African context. In African health systems, the imbalance between the demand for services and the resources available to meet those demands creates an environment that enables PSIs.^9^ Typical PSIs involve medication errors, wound infections, infusion reactions, pressure sores and falls.^10^ A patient safety culture requires teamwork, effective handover between teams, information exchange and open communication.^10^ The hospital survey tool on patient safety culture focusses on eight constructs: commitment to patient safety, priority given to patient safety, perception of the causes, investigation of PSIs, organisational learning following a PSI, communication of safety issues, staff education and teamwork.^11^ In Pretoria in 2019, a study across these eight constructs reported an overall positive safety culture, although 40% of individual health professionals had a poor culture.^11^ One study from Ghana in 2015 demonstrated the value of community engagement in reducing PSIs, particularly through improved leadership, accountability and staff competence.^9^

In the Western Cape’s district health services, clinical risk management and the PSI processes are part of clinical governance. Family physicians have clinical risk management and improving patient safety as part of their job description under clinical governance. Family physicians in the public sector of the Western Cape have formed a practice-based research network.^12^ The family physicians in this network identified a need to evaluate the implementation of PSI reporting and learning according to the guidelines. They ranked this knowledge gap and research question as their priority in November 2023. The aim was to evaluate the implementation of PSI reporting and learning in district health services of the Western Cape.

## Research methods and design

### Study design

This was a convergent parallel mixed methods study that collected both qualitative and quantitative data to measure a range of implementation outcomes (adoption, feasibility, fidelity, cost, reach, effects and sustainability) derived from Proctor’s implementation science evaluation framework.^13^ Mixed methods were needed to collect relevant data across all the outcomes at the same time. Quantitative data were downloaded from the PSI database on the Ideal Clinic digital reporting system. Qualitative data were collected in an exploratory, descriptive approach via semi-structured interviews.

### Setting

The study was conducted within a family physician practice-based research network within the Western Cape. The network was within the public sector district health services and included family physicians associated with both Stellenbosch University and the University of Cape Town. Family physicians worked at district hospitals and primary care facilities across all five health districts: Cape Metropole, West Coast, Cape Winelands, Overberg, Garden Route and Central Karoo. The Cape Metropole District was divided into four substructures: Khayelitsha-Eastern, Northern-Tygerberg, Southern-Western and Klipfontein-Mitchells Plain.

The directorate of quality assurance within the National Department of Health introduced a guideline for PSI reporting and learning in 2018. This was prompted by a national survey of hospitals that showed substantial variation and inconsistency in approach to PSIs. The guideline adopted the WHO international classification for patient safety. Compliance for a health facility was defined as submitting a monthly report. An analysis of the reporting system led to a revised guideline in 2022 and the launch of an online educational course.

### Study population, sample size and sampling

The study population consisted of 14 district hospitals and 12 primary care facilities which had a family physician who was a member of the practice-based research network. All these facilities were invited to provide their PSI data. In addition, one key informant was purposefully selected per facility. Key informants included eight family physicians, six quality assurance managers, six clinical or facility managers and six other clinicians (nurse practitioners or medical officers). These were also divided evenly between district hospitals and primary care facilities. A matrix was drawn up to link each family physician with one key informant at a different facility. Family physicians were linked to other facilities as they agreed to participate.

### Data collection

Each family physician downloaded the PSI data for their facility from the digital platform and provided it as an Microsoft (MS) Excel sheet. An interview guide was drafted by the first author (R.J.M.) and validated by the family physicians within the network. The guide had a broad opening question: *‘How well has the National Guideline for Patient Safety Incident Reporting and Learning in the Health Sector of South Africa been implemented at your facility?’* Following the opening question the implementation outcomes were explored using open questions listed in the guide. Family physicians were orientated to the guide in a virtual workshop and were trained in the communication skills needed for qualitative interviewing, and many had prior experience with such interviewing.

Interviews were held virtually and lasted between 30 min and 60 min. They were recorded using video conferencing software and were conducted in English, the official language of the health services.

### Data analysis

The MS Excel spreadsheets from each facility were collated into one MS Excel spreadsheet and irrelevant fields were deleted (e.g., personal identifiers and text). The Excel spreadsheet was then imported to the Statistical Package for Social Sciences version 27. Descriptive analysis reported on frequencies and percentages for categorical data. Numerical data were reported on as means and standard deviations or medians and interquartile ranges (IQRs), depending on their distribution.

The virtual recordings were transcribed verbatim by a professional transcriber. The first author (R.J.M.) checked each transcript against the original tape and made corrections. The data were then analysed thematically using the framework method and Atlas-ti. There was an overarching deductive framework based on the implementation outcomes:^14^

- Step 1: Familiarisation: A sample of the data was used to identify issues that could be coded.
- Step 2: Coding index: The researcher defined codes based on step 1 and organised them into categories.
- Step 3: Coding: The researcher applied the coding index to all the transcripts. If necessary, new codes were added.
- Step 4: Charting: The researcher created code families and brought all the data together from one category. A report was downloaded for each family containing all the data.
- Step 5: Interpretation: The researcher interpreted the data in each report to identify themes and subthemes. Any relationships between the themes were also noted.

### Trustworthiness

Trustworthiness for the qualitative data and analysis can be considered in terms of credibility, dependability, confirmability and transferability.^15^ The methods as a whole should be considered when evaluating these criteria, but some additional aspects are outlined here.

The analysis was presented to the family physicians in the network in a face-to-face workshop so that they could validate the interpretation and enhance credibility. In terms of confirmability, R.M. was an experienced qualitative researcher and full-time academic at Stellenbosch University. He helped to develop postgraduate training on clinical governance but was not involved in the implementation of the national PSI guidelines.

The family physicians were orientated to the study in a workshop, particularly the need for adherence to the interview guide, qualitative interviewing skills and reflexivity. They were aware that their own assumptions and beliefs should be bracketed during the interviews to enable the person to express their own experience and perspective openly and honestly. Their knowledge of the context and health services enabled more in-depth interviews.

### Ethical considerations

The study received ethical approval from the Health Research Ethics Committees at Stellenbosch University (reference no.: N24/02/015) and the University of Cape Town (reference no.: 295/2024). Permission to conduct the study was given by the Department of Health and Wellness.

## Results

Fifteen key informants were interviewed as shown in Table 1. Ten were from district hospitals and five from primary carefacilities, while eight were from Metro Health Services and seven from Rural Health Services. They included six family physicians, two facility managers, one nurse manager and three quality assurance managers, as well as two medical officers and a nurse practitioner. Nine were members of the committee dealing with PSIs and six were not.

**TABLE 1:** Characteristics of the key informants.

Number	Age (years)	Gender	Facility	District or substructure	Role	Years in post	PSI committee
1	43	Male	DH	CWD	Nurse manager	5.0	No
2	47	Male	DH	GRD	Family physician	3.0	No
3	45	Female	DH	CWD	Family physician	2.0	Yes
4	43	Male	DH	SWSS	Family physician	0.5	Yes
5	28	Female	PC	KESS	QA manager	2.0	Yes
6	51	Female	PC	KESS	Facility manager	9.0	Yes
7	44	Female	PC	KMPSS	Family physician	2.0	No
8	32	Female	DH	GRD	Medical officer	5.5	No
9	50	Female	DH	CWD	Facility manager	3.0	Yes
10	42	Female	DH	KESS	Medical officer	9.0	No
11	50	Female	PC	NTSS	Nurse practitioner	7.0	No
12	43	Female	PC	KESS	QA manager	2.0	Yes
13	42	Female	DH	SWSS	Family physician	5.0	Yes
14	41	Male	DH	WCD	Family physician	8.0	Yes
15	63	Female	DH	OBD	QA manager	15.0	Yes

PSI, patient safety incidents; DH, district hospital; PC, primary care facility; CWD, Cape Winelands District; GRD, Garden Route District; SWSS, Southern-Western Substructure; KESS, Khayelitsha-Eastern Substructure; KMPSS, Klipfontein-Mitchells Plain Substructure; NTSS, Northern-Tygerberg Substructure; WCD, West Coast District; OBD, Overberg District; QA, quality assurance.

### Reach of the patient safety incident reporting and learning system

**FIGURE 1:**
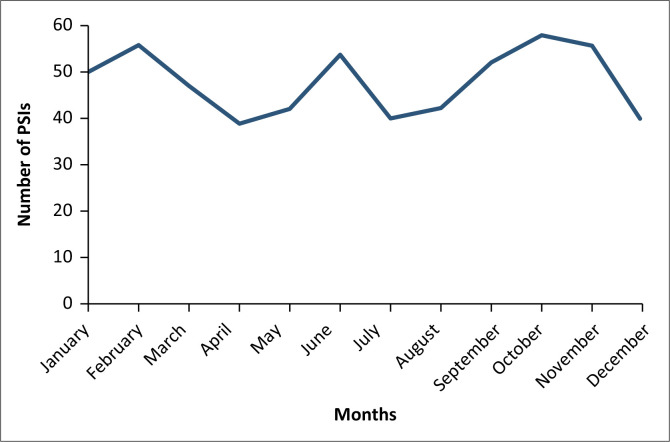
Distribution of patient safety incidents by month.

Sixteen facilities provided data on 577 PSIs during 2023 and the distribution of PSIs per district is shown in Table 2. Overall, 524 (90.8%) of the PSIs were from district hospitals and 53 (9.2%) from primary care facilities. The distribution of PSIs across the year is shown in Figure 1 and the median number of PSIs per month was 48 (range 40–58). The number of PSIs reported per facility was a median of 19 (range 0–148).

**TABLE 2:** Distribution of patient safety incidents across districts.

District	Number of facilities	PSI frequency	PSI %
Metro Health Services	9	154	26.7
Overberg	2	135	23.4
Cape Winelands	2	43	7.5
Garden Route	2	192	33.3
West Coast	1	53	9.2

PSI, patient safety incidents.

One primary care facility in the Metro had no PSIs reported at all in 2023. Feedback from the family physician on this outlier suggested that the reasons for this were staff missing the training because of shortages, oversight of the PSI process being located at the substructure and not the facility, loss of the PSI champion with inadequate handover and uncertainty about the definition of a PSI. All of this led to a lack of knowledge, skills and motivation among the staff regarding reporting PSIs.

### Adoption of the guidelines

All respondents articulated the importance of patient safety and working hard to prevent PSIs. They also emphasised the importance of adopting an open constructive process that did not attribute blame or seek to punish people. The PSI process was seen as potentially important in terms of reducing reputational risk to facilities in the local community, being accountable to individual patients and their families, avoiding future litigation, advocating for resources with higher management, reducing complications and hospitalisation, and improving the work environment. While all respondents resonated with these principles of working for patient safety, at least one saw the PSI reporting as a bureaucratic exercise that added little value, while another saw it as the most important meeting in the hospital. The former was also worried that if a facility reported more PSIs then this could be seen as a negative reflection on that facility rather than a successful adoption of the PSI guidelines:

‘So, I do understand it, but it’s just irritating, and because of that, I kind of need to be nudged by someone to do it. So I’m not overly keen to do paperwork that isn’t going to benefit me because the work has already been done and the case is resolved and sorted out, and now I must report to somebody, and I don’t think I see the relevance at my level of reporting it.’ (Family physician, PHC, MHS)

‘So where we, it’s probably one of the, it’s probably the most important meeting in our hospital. We’ve clarified that quite a couple of times. So apart from the hospital management meeting that summarises all the other stuff, the clinical risk management meeting is very important. It probably ranks like right at the top.’ (Family physician, DH, MHS)

Respondents believed that clinical staff saw the initiation of a PSI as a potentially punitive experience that could attribute blame or lead to disciplinary action. Clinicians might prefer to deal with the issue informally. There was a sense that district hospitals were moving towards a more open and engaged process of reporting and discussing PSIs when compared to primary care. Some respondents believed that the system for nurses might be experienced as more punitive than for doctors. In some facilities implementation of the PSI process has led to less concern from clinical staff over time. Nevertheless, respondents also reported that overzealous managers could blame and alienate staff in their desire to have the best facility:

‘There’s a desire to be the best clinic and in doing that, potentially alienating some staff from reporting, because the atmosphere is not necessarily one of, well we’re going to make mistakes. We’re going to, there are things that are going to happen. How are we best going to work through these problems, rather the attitude is we mustn’t make mistakes.’ (Family physician, PHC, MHS)

‘Yes. I know even at our facility, I know there have been doctors saying that we have weaponized the patient safety incident. They feel as if, like you mentioned, we want to target this specific person or this and in actual fact, it’s the situation that one wants to improve and yeah, and not necessarily the unfortunate nurse that did it.’ (Medical officer, DH, RHS)

There was an awareness of the guidelines, although few respondents had read them completely. Some had dipped in to check specific definitions, for example of different types of incidents. Awareness appeared higher among the senior managers and leadership, and much less among the clinical staff. Clinical staff might be aware of the forms to complete, but not the underlying guideline. Awareness appeared higher in the district hospitals than in primary care facilities, maybe because there were more issues with patient safety. People had some difficulty distinguishing between the old and new versions of the guideline:

‘Umm, I’ve browsed through them.’ (QA manager, SS, MHS)

‘I didn’t actually think about, oh, but there must be an actual guideline that each and every one of us can look at. So no, it’s not, it’s not available readily … If you have to go read a guideline, it will be the latest Obs and Gynae Guideline.’ (MO, DH, RHS)

District hospitals appeared to have more clearly defined committees, although the focus on PSIs was often incorporated into meetings that focussed more broadly on quality assurance or risk management (including infection prevention and control, occupational health and safety, quality of care and patient complaints). In at least one hospital it was combined with the morbidity and mortality meeting. In primary care, there was sometimes no committee, or the committee functions were coordinated from a substructure level or as part of the head of department meetings. Everywhere there was a need to balance the obligations to have meetings with the effects of taking people out of service delivery.

Membership of the PSI committee depended on how the committee was integrated with other meetings. The facility managers and family physicians were almost always involved, along with the quality assurance (QA) manager, if there was one. The other members of the committee varied but could include operational managers, pharmacists, human resource personnel, allied health professionals, supply chain managers, dental assistants and security or information management officers. Patient or community representatives were not part of these committees. The committee could be chaired by the family physician, QA manager, nursing or facility manager:

‘I am the chairperson of the forum. The other three specialists [*are the*] head of nursing, the different operational managers [*or*] nursing managers in their areas, [*and*] we have the quality assurance manager. The quality assurance manager actually deals with a whole host of things. So under her portfolio there is infection prevention control the OSS, ideal hospital, dealing with complaints, risk management and PSI … The only person we don’t have is a hospital board member, which is a requirement from the national guidance as well.’ (Family physician, DH, MHS)

Most facilities considered the PSIs in a monthly meeting, some met every 2 months or even quarterly, while one had PSIs on the agenda of every weekly head of department (HOD) meeting. Several respondents were unsure of how often the PSI committee met:

‘So we meet at our substructure, where everyone comes out to us every month and then when we go out to them at every second month.’ (QA manager, SS, MHS)

‘So it’s supposed to happen kind of quarterly … But then there have been times when it hasn’t. So no, it doesn’t happen regularly enough.’ (Facility manager, DH, RHS)

The functionality of the meetings varied. At one end of the spectrum, the meetings were used to just report on the number of PSIs and outcomes, while at the other end, meetings were used to analyse and discuss the incidents and brainstorm solutions. Sometimes this was more the function of the investigative team. When the PSIs were part of a larger meeting with multiple purposes then there was a tendency to report and not discuss in depth:

‘So often when there is a problem for the one matron and then the other matron might have a solution or something that she’s done from before that that she could give input in and often also from them more, umm, wider multidisciplinary or the sister in charge, they would be able to give solutions because of the experience from where they come from.’ (Family physician, DH, RHS, with a monthly PSI committee meeting)

‘We must discuss them at the meeting, but that’s more usually a bit perfunctory, like we had so many patient safety incidents, the themes were da, da, da, da, da.’ (Facility manager, DH, RHS, with a quarterly QA meeting)

### Feasibility and fidelity to the patient safety incident reporting and learning system

#### Type of patient safety incidents

The location of PSIs is shown in Table 3. Overall, 69.1% of reported PSIs reached the patient but only 33.5% were reported as causing harm. Serious harm or death was caused in 17.9% of the PSIs (SAC1) and of these 76.7% were because of patient behaviour. Patient behaviour was predominantly related to absconding from the facility. Accidents were mostly related to falls from the bedside. Medication issues were mostly related to prescription errors, omission of doses or medication, adverse drug reactions and incorrect medicines given. Clinical processes were mostly related to procedures, followed by errors in clinical assessment. The main contributing factors were patient behaviour, issues with language and communication, as well as social determinants such as living conditions, social support and education.

**TABLE 3:** Type and location of patient safety incidents (*N* = 577).

Type of behaviour	Frequency (*n*)	%
At-risk behaviour	141	24.5
Human error	164	28.5
No error	235	40.8
Reckless behaviour	36	6.3
**Type of incident**		
Harmful	193	33.5
Near miss	178	30.9
No harm	205	35.6
**SAC score**		
SAC1	103	17.9
SAC2	143	24.8
SAC3	188	32.6
SAC4	142	24.7
**Location**		
Hospital wards	416	72.2
Emergency centre	45	7.8
Maternity ward or unit	31	5.4
Psychiatric services	30	5.2
Primary care	12	2.1
Pharmacy	8	1.4
Operating theatre	8	1.4
Outpatients	6	1.0
Other	20	3.5
**Main class of PSI**		
Patient behaviour	136	23.6
Patient accident or fall	120	20.8
Medication or IV fluids	100	17.4
Clinical processes	85	14.8
Nosocomial infection	23	4.0
Clinical administration	18	3.1
Staff behaviour	10	1.7
Infrastructure	9	1.6
Medical equipment	9	1.6
Other	60	10.4
**Contributing factors (*N* = 259)**		
Patient behaviour	153	59.1
Patient communication	70	27.0
Patient social	68	26.3
Infrastructure	22	8.5
Security and safety	20	7.7
Equipment and products	7	2.7
Staff social	6	2.3
Equipment	6	2.3
External providers	4	1.5
Consumables	3	1.2
Environmental risk	2	0.8
Teamwork	2	0.8
Natural event or disaster	2	0.8
Regulations	1	0.4
Other	14	5.4

IV, intravenous; PSI, patient safety incidents; SAC, severity assessment code.

Most facilities reported the PSIs that caused actual harm and did not focus on incidents of potential harm:

‘The no harm ones, we haven’t, I don’t think we even thought of reporting them. I don’t know, we did have those ones.’ (Facility manager, PHC, MHS)

District hospitals had a particular problem with patients who had acute psychiatric problems. Safety incidents arose from inadequate infrastructure to separate and monitor these patients. This resulted in patients absconding, attacking staff or other patients, damaging equipment, developing complications or harming themselves. Adolescent psychiatric patients were particularly vulnerable to PSIs:

‘There was actually a patient that burned the whole ward down just in the time that I was there and almost burned a non-psych patient.’ (Family physician, DH, RHS)

‘We don’t have a dedicated psychiatric unit, so we have to move the psychiatric patients around to make beds and sometimes more than once a day for other patients. And because of the pressure and maybe the cleaning, not happening so well, they developed these nosocomial pneumonias.’ (Family physician, DH, RHS)

‘So our most famous one was when two teenagers had sex in the mental health ward, and it was a statutory rape. So that was our biggest patient safety incident in my time.’ (Facility manager, DH, RHS)

‘The one thing that keeps recurring are abscondments and the biggest group are psychiatric patients … Because you got three exit points in our EC, makes it difficult for two security guards to actually manage.’ (Family physician, DH, MHS)

Incidents related to patients absconding were also reported for other types of patients:

‘The last SAC 1 would be this month, would have been a paediatric case where the mother absconded with the child from our paediatric ward.’ (Family physician, DH, MHS)

District hospitals highlighted a problem with falling out of bed because of faulty bedsides in elderly or confused patients. Primary care facilities also reported patients slipping and falling. Conversely, there were also issues with bedsores in immobile patients:

‘Patients who fell from the bed. And then the patient, nothing, nothing really is broken or fractures or anything that was sustained. But yeah, it is then for us to just be aware and see what is the reason, why does it happen now. Is it the cot side that that is broken or faulty?’ (Nursing manager, DH, RHS)

Maternity issues were another common source of PSIs, particularly in the midwife units attached to primary care facilities. Incidents were related to giving birth at the facility but outside the unit, incorrect assessment of antenatal risk, mistakenly giving misoprostol and retained products of conception:

‘[*S*]he was treated as low risk by the midwives until when she was pushing, when she was bearing down, then she said it was difficult for her, for her to push, then, then she said the she was diagnosed with cardiac. So then it was difficult. Then unfortunately, the baby died during that time because it took longer. It was stillborn.’ (Facility manager, PHC, MHS)

Several PSIs related to incorrect prescriptions, administration of schedule 6 drugs by junior staff, not giving the medication or IV fluids as prescribed, falsely reporting that a medication has been given, dispensing the wrong medication, not responding correctly to an adverse drug reaction or being unaware of medications obtained elsewhere. Many of these incidents that caused no obvious harm were not reported:

‘And we’ve been actively, you know, we actually had to teach the pharmacy and the support services to report the incidents that happens in their areas that did not yet cause harm, for examples would be like pharmacy has a lot of like incidents or mini-incidents that they would maybe not necessarily have reported before.’ (Family physician, DH, RHS)

Primary care facilities also highlighted issues in the emergency centres. There were issues related to the preparedness of emergency centres, such as stocking the emergency trolley or ensuring sufficient oxygen supply. There were also issues with patients deteriorating while waiting to be seen or having hidden weapons:

‘For instance, there was also a case, yes it was serious, but upon the investigation it was the issue of the oxygen that was in. There was no oxygen in trauma unit.’ (Nurse manager, PHC, MHS)

Other issues in primary care related to needle stick injuries, untruthful medical histories, poor follow-up of patients, lack of supplies and dental procedures. A few respondents thought that poor teamwork and patient handover in the wards or emergency centres were potential causes of PSIs:

‘They come and complain to me because of the staff members that is having issues with the handover of patients when they take them, maybe from the emergency centre. So I think when that happens then there’s maybe critical information getting lost. When staff members have issues with each other, or when somebody isn’t doing what they supposed to doing.’ (Nursing manager, DH, RHS)

#### Identification and reporting of patient safety incidents

The median time to report a SAC1 was less than a day (0.0; [interquartile range {IQR}: 0.0–1.0]), although 20.4% of SAC1s were reported after 2 days or more (range 0–188 days). Table 4 shows the main methods for reporting.

**TABLE 4:** Reporting methods for patient safety incidents (*N* = 259).

Reporting method	Frequency (*n*)	%
Health professional	253	97.7
Patient complaint	2	0.8
Inpatient medical review	1	0.4
Media	1	0.4
Initiated by province	1	0.4
Record review	1	0.4

PSI, patient safety incidents.

Most of the PSIs were reported by staff, usually doctors or nurses. However, there were issues with recognising that a PSI had occurred, and staff were frequently prompted by managers or the family physician to complete the form. Clinical staff worried about eroding the team relationships and getting someone into trouble if they reported a PSI. Patient safety incidents were often only reported when the manager became aware of the incident. Some reported that older staff were more reluctant to engage with the PSI process and new staff needed orientation and training:

‘And in most cases, you will hear in corridors that this has happened, but the staff member didn’t realize actually this is a PSI you know or when she as manager when she hears there was an incident that has happened, no actually it is a PSI so we must like report it.’ (Nurse manager, PHC, MHS)

A few respondents reported some confusion about the difference between types of events, for example PSIs versus adverse drug reactions. Respondents reported that PSIs were identified after a patient complaint or in mortality and morbidity meetings, although the quantitative data did not support this.

In a few facilities, the family physicians would identify more minor PSIs during their ward rounds. Serious PSIs that involved a death or significant harm were more easily identified, although incidents that occurred over weekends were often not reported in the 24-h window period:

‘And then the one thing that that I also have to say some of the SAC ones for argument’s sake. Now we have to report it within 24 hours, so we don’t really abide to that timeline. I think sometimes things happen. And then maybe 3–4 days later, then we will be able to report it because you have to investigate also and then specifically over weekends. Then none of the management are here.’ (Nursing manager, DH, RHS)

#### Investigating the patient safety incidents

Overall, the median number of days to close the PSIs was 4 days (IQR: 1.0–12.8) with a range from 1 to 77 days. It appeared that the intended steps of the PSI process were mostly followed, partly because the form itself was structured according to these steps. In one facility the forms were available in hardcopy and on the facility’s OneDrive. The details were also entered electronically into the Ideal Clinic system, which also guided people on the steps. People were not very aware of the underlying guidelines. For some, it was more of an administrative exercise than a genuine exploration and root cause analysis:

‘It’s a little bit difficult. So, the first thing that we focused really on was on reporting because initially the reporting just wasn’t happening. So that was very much the initial phase, which I think we’ve gotten right but we’re not doing that well in closing the loop, you know, doing all of it.’ (Facility manager, DH, RHS)

In most cases, the initial report was completed by a member of the clinical staff and then escalated to their operational manager. Sometimes the PSI committee members were then informed. The family physician was often involved in investigating the incident with the operational manager, after which the PSI was formally reported to the committee. The investigation required input from several people and could be delayed if they were on shift changes or reluctant to participate. Where there was a QA manager then they would coordinate the process and capture the report. In other places, information management or other senior staff had to capture the PSI electronically. In one facility with 5–8 PSIs discussed per month, they had to prioritise which PSIs were discussed fully before the meeting:

‘The medical officers here and now are also quite good in reporting so they will report the incident and also then report it on the PSI form and then they will give it also to the operational manager in in certain instances they will give it to the family physician, who will then give it to the operational manager and then after their initial investigation, it will come to me and then I will finalize the process and capture it.’ (Nursing manager, DH, RHS)

### Costs of implementing the patient safety incidents guidelines

Implementation of the process had no real incremental costs, although implementing the recommendations was constrained by financial resources, especially where this related to infrastructure or equipment:

‘Oh, some of the things that cost a lot, for instance, where we had the amount of people falling from their cots, but we found it was four beds that had broken side rails. And just because, due to costs, we couldn’t replace them.’ (Family physician, DH, MHS)

There were significant opportunity costs in the time taken to complete the forms, investigate the incidents, meet as a committee and provide feedback to staff or patients. In some facilities, the benefits to patient safety were seen as outweighing the impact on service delivery. Others struggled with the impact on patient care:

‘I think what I’ve calculated in my mind is that that an hour spent for the patient safety incidents is probably much more important than for instance, a family physician going to a clinic or because that is where you can pull everything together and make this a strategic plan to influence governance.’ (Family physician, DH, RHS)

‘And people get meeting fatigue, you know, especially to try and get your operational managers involved in a meeting like this. Especially if they are short staffed, it’s difficult. It’s difficult to take them out of their clinical areas and spend time, because a lot of other meetings we expect them to attend. So that’s the main issue is the time.’ (Facility manager, DH, RHS)

### Effects of the patient safety incidents guidelines

Some respondents saw the value of the PSI reporting process in advocating with higher management for resources to improve patient safety. The PSI process gave clear evidence for a particular change and could therefore be used as ammunition in motivating resources. The value of the PSI process to staff was in its ability to evoke change and improve safety, and this could also motivate staff to participate. Engaging staff to participate in decision-making, for example, the selection of new equipment, could also add value and motivate staff.

The current austerity measures and budget cuts were seen as placing even more constraints on the ability to implement recommendations. However, many of the recommendations were behavioural and required few financial resources. Staff shortages, turnover, motivation and involvement of locums or external stakeholders could hinder behavioural changes. Some respondents commented that simple fixes would probably be made anyway without the complicated PSI process.

The PSI committee could monitor trends and recurrence and follow up on whether action was taken. When the recommendations were within the locus of control of the facility, then usually they did not recur, but some were dependent on outside actors such as emergency medical services or external resources. Recurrent PSIs such as falls are often reduced over time with persistent attention:

‘Low hanging fruit. Try to get quick wins because it also boosts morale. Just if they can say that can see that something actually came from the PSI reporting. Now we’ve seen this change and it’s working. It’s helping to prepare that next PSI again.’ (Family physician, DH, MHS)

Most feedback to staff happened on a one-on-one basis or to the people directly involved in the unit. Often the operational manager was responsible for such feedback. Feedback to the staff in general was usually more a reporting on the overall statistics, if such feedback happened at all. One respondent felt that feedback to the doctors was experienced as constructive, while feedback to nurses was experienced as punitive. Most respondents felt more general feedback and engagement of staff would be helpful:

‘That is the gap, that is the gap because always like individually. We go to the individual person if it was the case of negligence or even in terms of SOP we give the SOP to that operational manager. But then to follow up if it was implemented or the staff is aware of it ja I think ja there is a gap in that area. So we don’t go back like call a staff meeting and present maybe our stats or our cases and recommendations like in a group, we don’t do that.’ (Nursing manager, PHC, MHS)

When the PSI caused harm or originated from a complaint then the patient or family members were informed about it and the recommendations:

‘So obviously with patients, if it is a very, if it’s a SAC 1 or SAC 2, that happens immediately. So we are very open with our patients. We have meetings with them. And we’ve learned a lot through our SAC 1 process, you know, just engaging and being open and honest with our patients.’ (Family physician, DH, MHS)

When the underlying problem was a shortage of staff and high workload, it was difficult to implement a solution because of austerity measures in the health system. During coronavirus disease 2019 (COVID-19), there had been more responsiveness to staffing issues. In a few cases, the problem could be solved by avoiding locum staff in a high-risk area:

‘So at the end of the day, you totally are aware that there’s a potential risk that certain things can happen. And then to put disciplinary measures in place or punish the people. I didn’t feel it’s fair. Because they tried their best, to ensure that, they were the minimum people do all the things that is required to be done and at the end of the day they unfortunately weren’t able. But then you go back and you look how many people was in the facility.’ (Nursing manager, DH, RHS)

It was also difficult to solve problems with equipment in the current financial climate. Occasionally equipment, such as beds or bedside bell systems, could be fixed on-site, but more complicated repairs were hindered by the unavailability of parts or long delays in making the repairs. In a few instances, the management committed the financial resources to buy new equipment such as burglar bars, cameras in the psychiatric unit, beds or air mattresses, with good results:

‘So yeah, I do think that at a cost with investing in new beds in mattresses this has paid off. So far, we haven’t had any falls from our general ward or any bed pressures reported so far.’ (Family physician, DH, MHS)

Infrastructure changes were crucial in addressing some of the challenges faced by psychiatric patients. The PSIs highlighted the necessity of these changes, but financial constraints limited what was achievable. In most cases, these changes were seen as a long-term strategy. However, the health system’s inability to respond to many of the recommendations discouraged people from investing time and energy in the PSI process:

‘We proposed that we use a ward that was not being used to create a 72 hour unit unfortunately even though that report went very high up, all the way up, they couldn’t find the money to support that, but then we did manage to install gates in the ward and remove all the you know the dangerous and infrastructural components for the psychiatric patients.’ (Family physician, DH, RHS)

Behavioural issues were the easiest to address within the facility. Respondents were aware of the relationship between workload and having to work efficiently at speed, with the increased likelihood of a PSI. A wide variety of examples were given, such as creating standard operating procedures, new checklists, tools to assess patient risk, communicating the importance of change or using a whiteboard to highlight key aspects of patient care:

‘The one example that I do know of is that, okay, I mentioned the fall, that some patients get PSIs done when they fall, and we implemented a new scoring sheet and printed those and the training on, for the nurses and the doctors for when to do that score and what does the score mean. So that was done. That’s what I remember was implemented.’ (Medical officer, DH, RHS)

Behavioural changes sometimes also involve the referral pathway. One facility reported the use of WhatsApp to communicate better over the need for referral of psychiatric patients:

‘So they can understand exactly when we say we can’t deal with four psychotic patients in one stage and why we struggle to deal with our psychiatric patients and why we have a high abscondment rate. So from there on, we did the greater WhatsApp group so they follow up on their team on as to how far they are with accepting our patients just to make the process go faster and smoother. So that should been happening the last two months.’ (Family physician, DH, MHS)

Implementing the recommendations once the PSI was captured electronically was often a problem, and not every facility followed up on the action plans to monitor implementation:

‘It’s always when they have to carry out the recommendations because when we do filing and whatever the recommendation there was, there has to be an action after that and then you have to file everything with that PSI. So the hiccup is always related to when the recommendation has to be carried out.’ (QA manager, PHC, MHS)

### Sustainability of the patient safety incident reporting and learning system

Several respondents thought that talking about PSIs in regular meetings would help to change the culture and make the process less threatening. In primary care, PSIs were less frequent, and people needed to be aware of the possibility. Respondents also believed that regular training was necessary to motivate and inform staff, particularly in high-risk areas and with staff turnover. Specifically training all the relevant facility and operational managers could transform the attitude towards PSIs and an online training course was available on the knowledge hub. This led to a change in organisational culture, with less blaming and more understanding of the steps involved. This also helped people to distinguish between PSIs, clinical risk management and morbidity and mortality issues, and led to more PSIs being reported each month:

‘So initially I found myself very, very, yeah, unequipped. But the knowledge hub came up with this training online and I did it initially at first and then I taught my team through it and then I actually had it mandated by our hospital manager to include it in all the PAs of the managers in the hospital.’ (Family physician, DH, MHS)

Several facilities did not have QA managers and responsibility fell on the shoulders of other managers. The QA manager coordinated the steps of the PSI process, supported the PSI committee and captured the process electronically. These posts had not been filled because of austerity measures. Quality assurance managers, however, did not actually do the investigation themselves. Most facilities with QA managers reported a much smoother PSI process, although one facility that had trained all the managers thought the QA manager was not necessary. However, even there the uploading of PSIs on the Ideal Clinic system was an issue:

‘You are the person that must now sometimes investigate or put the final comments and then also have to capture. Then it becomes a lot of work, especially when everybody is sending the things to you in the absence of the QA [*manager*] now and you have to juggle this with all the other duties that that you are now expected to do, so that is the only issue for me.’ (Nursing manager, DH, RHS)

One respondent commented on how the forms were hospital-centred, cumbersome and not tailored to the primary care context:

‘Yeah, I do find the form not very user-friendly, and that probably is part of why I also think this is going to be 10–20 minutes of my life, which is not actually that beneficial to anybody, and it’s cumbersome and not that great to use.’ (Family physician, PHC, MHS)

The current austerity measures and shortages of staff were driving PSIs and so this needed to be reversed if the PSI process was to be successful:

‘The other resources, it’s really difficult because we can’t fill posts. So we’ve got two operational manager posts that are vacant, and we’ve got seven professional nurse posts that are vacant in the hospital. You know, so it’s very, very difficult to focus on quality and patient safety when you actually don’t have nurses, you don’t have managers.’ (Facility manager, DH, RHS)

## Discussion

### Summary of key findings

**FIGURE 2:**
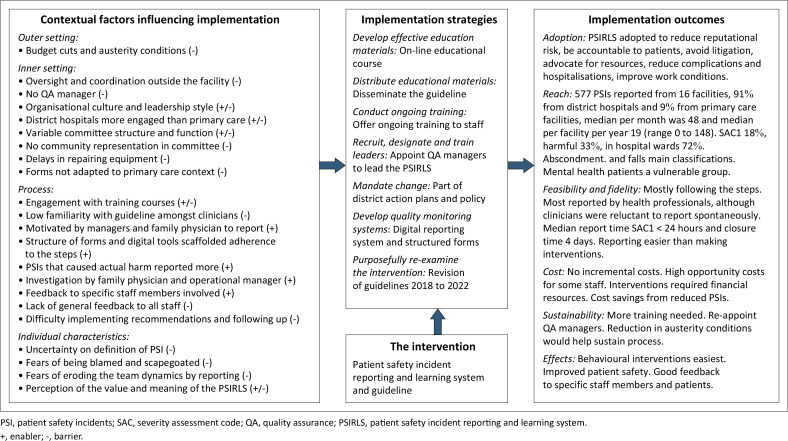
Summary of key findings.

The key findings are summarised in Figure 2 as an implementation research logic model. The implementation outcomes and contextual factors are derived from the findings. The implementation strategies are identified from the guidelines and feedback from participants.

### Discussion of key findings

The PSIRLS in the Western Cape appears to be fulfilling most of the expected functions^16^: communicating risks to the healthcare facility and alerting the health system, acting as a barometer for the level of risk prevalent in facilities, and providing a foundation for learning and improvement. There was less evidence that the system was responding to the concerns of patients and families or providing transparent, independent accountability to the public. The number of PSIs reflected engagement with reporting more than the risk prevalent in facilities.

The success of the PSIRLS depends partly on the organisational culture. Most managers and family physicians had a positive attitude towards the system, while clinicians appeared to be less aware and more concerned about blame. There may be a risk of a second victim syndrome if healthcare workers are made to feel personally responsible for PSIs and they may also require psychological support.^17^ Training all the managers on the PSIRLS at the one facility appeared to transform the culture. Implementing the system with a focus on learning and improvement also appeared to change attitudes over time. The need to transform the leadership style has been noted in evaluations of the organisational culture,^18^ and it is possible that nursing has a more authoritarian approach.

The reputational risk to health facilities of PSIs was recognised by at least one key informant. In 2024, South Africa passed the National Health Insurance (NHI) Bill and is committed to the transformation of the health system.^19^ Reassuring the public that public sector healthcare is safe and of high quality will be a key ingredient in building public trust in the implementation of NHI.

Contributing factors were mostly attributed to patients and this may indicate the need for engaging patients and improving health literacy as well as overcoming language barriers. These activities have been found to decrease harm.^9^ Unfortunately, no respondents spoke of engaging patient representatives, clinic committees or hospital boards in the PSI process. Community engagement is a key ingredient in the provincial commitment to community-orientated primary care,^20^ but is yet to be fully realised. Most facilities however were committed to disclosure of PSIs to patients and families. Globally only 25% of countries have procedures for such disclosure.^2^

Several respondents reported that infrastructural and equipment-related interventions were not possible because of austerity and budget restrictions. One or two facilities had planned such expenditures with good results, despite restrictions. There is good evidence, however, that PSIs are a major contributor to expenditure through additional medical interventions and the use of resources.^3^ It may therefore be short-sighted to delay such interventions because of cost. In addition, this equation does not consider the indirect costs for patients and society through loss of income and productivity, which may be even higher.

There was less engagement of primary care facilities in both the research and the PSI process. This could be because of fewer PSIs, but more likely to under-reporting that might be related to the organisational culture, lack of training and fear of reporting. This difference was also reflected in the smaller proportion of PSIs reported in the Metro (eight facilities, 27% PSIs) versus rural health services (seven facilities, 73% PSIs). Globally, there has been less priority given to safety in primary care and only 17% of countries included primary care in the processes.^2^

### Strengths and limitations

Only 15 out of a potential 26 facilities provided qualitative data. However, data were collected from hospitals across all districts and the primary care facilities included all substructures in the Metro. Overall, therefore, the interviews had a good geographical spread across the province. Family physicians and managers were well covered in the data, but only three other clinicians gave input. District hospitals contributed more data than primary care facilities, which may reflect a greater commitment to the evaluation of PSIs in the former group. Two different types of PSI datasets were received and thus data were not available from all facilities for every variable. Hence the reduced denominator for some results.

### Implications

Training all the managers and family physicians in the PSIRLS could improve implementation and change the organisational culture. All clinical staff (e.g. nurses, doctors and pharmacists) should be informed of the guidelines and PSIRLS during induction or continuing professional development. Regular feedback on PSIs would assist with this. Postgraduate training of family physicians should ensure that patient safety and risk management are emphasised as part of clinical governance.

Particular attention should be given to improving the infrastructure of district hospitals to cope with the large numbers of patients with acute psychiatric problems who require 72-h observation. Substance-related acute psychosis is a major problem in the Western Cape.^21^

Managers should be encouraged to use their budgets to improve equipment and supplies that contribute to PSIs. The increased expenditure from patient harm is likely to outweigh the expenditure to prevent such harm. For example, bedsides should be fixed as a priority issue.

Re-appointing QA managers would reduce the administrative burden on other managers and family physicians and ensure the PSIRLS is coordinated and monitored.

Consideration should be given to adapting the PSIRLS more to primary care and not just the hospital context. It might also be possible to integrate some of the reporting and reduce bureaucracy when an incident requires reporting in other channels such as via the *Mental Health Care Act* or as an adverse drug reaction.

Thought should be given to involving patient representatives more in the PSIRLS and making the system more transparent and accountable to the public.

## Conclusion

A digital PSIRLS has been adopted and implemented in the Western Cape; however, few people have read the guidelines, resulting in wide variation in reporting levels. Many PSIs were related to patients absconding, particularly those with acute psychiatric issues, as well as incidents of falls. Clinical staff remain concerned about the potential for blame and the erosion of teamwork resulting from reporting. Behavioural interventions were the easiest to implement, while issues related to infrastructure, equipment, supplies and workforce were constrained by current austerity measures. Family physicians and managers led the process and recognised the value of having clear evidence to support improvements. Facilities should ensure that all leadership is trained in the PSIRLS and provide regular feedback to all staff. Greater attention should be given to engaging patients and enhancing accountability to the public.

## References

[1] 72nd World Health Assembly. Global action on patient safety WHA72.6 [homepage on the Internet]. New York, NY; 2019 [cited 2025 Jan 10]. Available from: https://apps.who.int/gb/ebwha/pdf_files/WHA72/A72_R6-en.pdf

[2] World Health Organization. Global patient safety action plan 2021–2030: Towards eliminating avoidable harm in health care [homepage on the Internet]. Geneva; 2021 [cited 2025 Jan 10]. Available from: https://www.who.int/teams/integrated-health-services/patient-safety/policy/global-patient-safety-action-plan

[3] Slawomirski L, Auraaen A, Klazinga N. The economics of patient safety: Strengthening a value-based approach to reducing patient harm at national level: OECD Health Working Paper No. 96 [homepage on the Internet]. Paris; 2018 [cited 2025 Jan 10]. Available from: 10.1787/5a9858cd-en

[4] WHO: 10 facts on patient safety (September 2019) – WHO – Patient safety learning – The hub [homepage on the Internet]. [cited 2024 Feb 01]. Available from: https://www.pslhub.org/learn/organisations-linked-to-patient-safety-uk-and-beyond/international-patient-safety/who/who-10-facts-on-patient-safety-september-2019-r557/

[5] National Department of Health. National guideline for patient safety incident reporting and learning in the health sector of South Africa. Pretoria: National Department of Health; 2022.

[6] World Health Organization. Global patient safety report 2024. Geneva: World Health Organization; 2024.

[7] Connell L. A clinical governance handbook for District Clinical Specialist Teams. Durban: Health Systems Trust; 2014.

[8] Mash R, Blitz J, Malan Z, Von Pressentin K. Leadership and governance: Learning outcomes and competencies required of the family physician in the district health system. S Afr Fam Pract. 2016;58(6):232–235. 10.1080/20786190.2016.1148338

[9] Alhassan RK, Nketiah-Amponsah E, Spieker N, et al. Effect of community engagement interventions on patient safety and risk reduction efforts in primary health facilities: Evidence from Ghana. PLoS One. 2015;10(11):e0142389. 10.1371/journal.pone.0142389 26619143 PMC4664410

[10] Poku CA, Attafuah PYA, Anaba EA, Abor PA, Nketiah-Amponsah E, Abuosi AA. Response to patient safety incidents in healthcare settings in Ghana: The role of teamwork, communication openness, and handoffs. BMC Health Serv Res. 2023;23(1):1072. 10.1186/s12913-023-10000-0 37803364 PMC10559624

[11] Bongongo T, Govender I, Olowa SN, Phukuta NSJ, Nzaumvila DK. Level of patient safety culture among public healthcare professionals in Pretoria. S Afr Fam Pract. 2023;65(1):5640. 10.4102/safp.v65i1.5640 PMC10244935 37265136

[12] Mash R. Establishing family physician research networks in South Africa. S Afr Fam Pract. 2020;62(1):1–4. 10.4102/safp.v62i1.5216 PMC8378057 33179955

[13] Proctor E, Silmere H, Raghavan R, et al. Outcomes for implementation research: Conceptual distinctions, measurement challenges, and research agenda. Adm Policy Ment Health. 2011;38(2):65–76. 10.1007/s10488-010-0319-7 20957426 PMC3068522

[14] Ritchie J, Spencer L. Qualitative data analysis for applied policy research. In: Bryman A, Burgess R, editors. Qualitative data analysis. London: Routledge, 1994; p. 173–194.

[15] Lincoln Y, Guba E. Naturalistic inquiry. London: Sage Publications; 1985.

[16] World Health Organization. Patient safety incident reporting and learning systems: Technical report and guidance. Geneva: World Health Organization; 2020.

[17] Nzaumvila D, Bongongo T, Govender I, Okeke S. An evaluation of support to the second victims in Tshwane District Health Services, South Africa. S Afr Fam Pract. 2024;66(1):a5980. 10.4102/safp.v66i1.5980 PMC11447557 39354789

[18] Gilson L, Daire J. Leadership and governance within the South African Health System. In: Padarath A, English R, editors. South African health review. Cape Town: Health Systems Trust, 2011; p. 69–80.

[19] Government of South Africa. Act No, 20 of 2023: National Health Insurance, Act 2023 [homepage on the Internet]. 50664 South Africa; 2024 [cited 2025 Jan 10]. Available from: https://www.parliament.gov.za/storage/app/media/Acts/2023/Act_20_of_2023_National_Health_Insurance.pdf

[20] Western Cape Government Health and Wellness. Western Cape Government health position statement on community-oriented primary care (COPC) towards universal health coverage (UHC). Report No.: Circular H11/2023. Cape Town: Department of Health and Wellness; 2023.

[21] Plüddemann A, Dada S, Parry C, et al. Monitoring the prevalence of methamphetamine-related presentations at psychiatric hospitals in Cape Town, South Africa. Afr J Psychiatry. 2013;16(1):45–49. 10.4314/ajpsy.v16i1.8 23417636

